# Validation of an Endoscopic Landmark for Injection of Internal Branch of Superior Laryngeal Nerve

**DOI:** 10.1002/lary.70050

**Published:** 2025-08-19

**Authors:** Yuki Tanigami, David Hortobagyi, Andrew J. Bowen, Chelsea Kruse, Seth H. Dailey

**Affiliations:** ^1^ Division of Otolaryngology‐Head and Neck Surgery, Department of Surgery University of Wisconsin School of Medicine and Public Health Madison Wisconsin USA; ^2^ Department of Otorhinolaryngology–Head and Neck Surgery Medical University of Vienna Vienna Austria; ^3^ Metrohealth Medical Center Cleveland Ohio USA

**Keywords:** 3D‐printed model, internal branch of superior laryngeal nerve, laryngeal sensory dysfunction, transnasal injection

## Abstract

**Objective:**

This study aims to validate a novel transnasal endoscopic approach for accurately targeting the internal branch of the superior laryngeal nerve (iSLN) via the piriform sinus, with the goal of improving treatment outcomes for laryngeal sensory dysfunction (LSD).

**Methods:**

Eight cadaveric larynges were examined using a 3D‐printed cadaveric laryngeal model. The superolateral quadrant of the piriform sinus was endoscopically marked with toluidine blue ink. The distance between the ink mark and the iSLN confluence point was measured as well as other landmarks.

**Results:**

The mean distance from the ink to the iSLN confluence was 5.38 mm, with significantly less variability than traditional external landmarks.

**Conclusion:**

The transnasal approach provides a reliable, anatomically precise method for accessing the iSLN, offering potential advantages over traditional external techniques in the management of LSD.

## Graphical abstract

This study validates a transnasal endoscopic approach to target the internal branch of the superior laryngeal nerve (iSLN) via the piriform sinus. Cadaveric analysis showed this method enables precise localization with minimal variability, offering a promising technique for treating laryngeal sensory dysfunction.

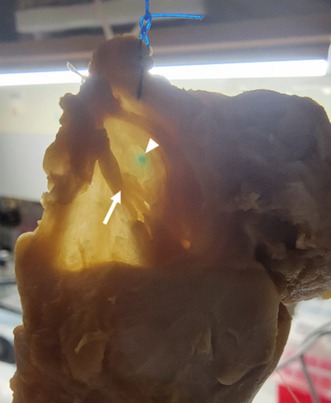

## 1 Introduction

Unexplained chronic cough is a challenging clinical entity to treat [1]. Options include systemic medications such as amitriptyline, gabapentin, lyrica, and others; many have demonstrated successful suppression of cough, but side effects such as dry mouth, sedation, and dizziness in the setting of ongoing administration can be barriers to ongoing use [2]. Speech therapy has also proven effective but requires numerous patient visits and ongoing compliance to the strategies, making long‐term results questionable [1]. In 2018, Simpson et al. introduced the office‐based technique of external needle delivery of steroids and lidocaine to the internal branches of the superior laryngeal nerve [3]. This technique uses anatomic landmarks to guide delivery of the medication to the area of the iSLN as it exits the thyrohyoid membrane. Numerous follow‐up studies show favorable short‐term suppression of cough and other suspected neurosensory laryngeal disorders such as chronic throat clearing, globus pharyngeus, and paradoxical vocal fold motion [4, 5, 6]. A follow‐up injection study also shows that bilateral simultaneous injection is safe and efficacious [7]. The short‐term efficacy of this technique, however, remains a limitation to wide acceptance. One possible reason for limited duration of effect is anatomic inaccuracy of delivery based upon the external approach; anatomic landmarks may be obscured due to prior neck incisions with scar, obesity, or other reasons. When true, one might anticipate a technical “miss,”

To address this anatomic limitation, we have undertaken a transnasal approach in the office using a working‐channel laryngoscope and topical anesthesia to inject therapeutic medication immediately next to the iSLN in the hypopharynx [8]. This transnasal approach takes advantage of well‐described anatomic descriptions of the iSLN as it courses submucosally in the thyrohyoid space [9]. We further hypothesize that optimized cough suppression will depend upon accurate delivery of medication to the area of confluence of the inferior, middle, and superior small branches of the iSLN as they become the iSLN proper [10]. To achieve delivery at that site endoscopically, we have been injecting the superolateral quadrant of each piriform sinus. We now seek to assess the veracity of our quadrant choice using a hybrid model with a 3‐D printed head and neck and human cadaveric larynges and real‐world instruments.

## 2 Materials and Methods

### 2.1 Study Design and Specimens

This study was conducted using eight human cadaveric specimens. The laryngeal specimens were preserved in formalin. To be included in the study, specimens were required to have an intact larynx, including the hyoid bone. The endolaryngeal and pharyngeal mucosa, particularly within the piriform sinuses, also needed to be intact. Additionally, at least three tracheal rings were required to remain attached to the larynx.

IRB approval for this was not needed given that the cadaveric specimens had been donated and were entirely deidentified.

### 2.2 3D Printing

A novel 3D‐printed model served as a framework to facilitate the insertion of a flexible endoscope via the nasopharynx, providing a realistic view of the larynx and hypopharynx from above. The design was based on data from a publicly available CT scan of the head and neck, obtained as an .stl file from the National Institutes of Health (NIH) 3D repository (https://3d.nih.gov/entries/3DPX‐004646). The 3D model was printed using a Phrozen Sonic Mega 8 K resin 3D printer (Phrozen, Hsinchu, Taiwan), utilizing ANYCUBIC standard 3D printer resin (ANYCUBIC, Shenzhen, China).

### 2.3 3D Hybrid Model Setup

**FIGURE 1:**
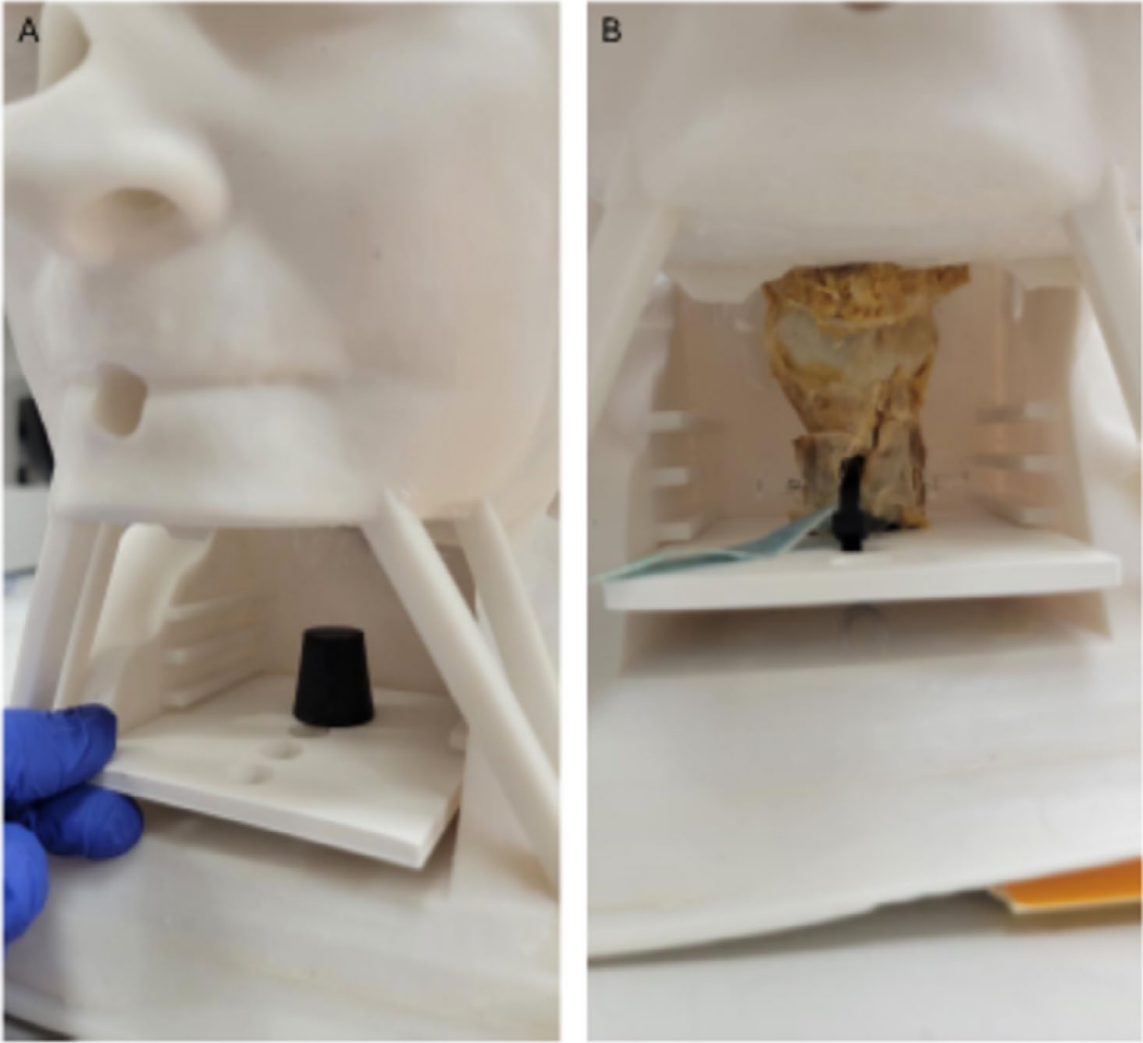
3D model from outside without (A) and with (B) cadaveric larynx.

Prolene 2–0 sutures were attached to the epiglottis and the lateral pharyngeal walls. A cork, mounted on a 3D‐printed plate, was inserted into the trachea and secured with pins. A plate functioned as a drawer system, fitting into an anterior window in the neck (Figure 1). The Prolene sutures were threaded through designated openings on the anterior and lateral sides of the model, allowing for tensioning to simulate the in vivo anatomy of the larynx and hypopharynx.

**FIGURE 2:**
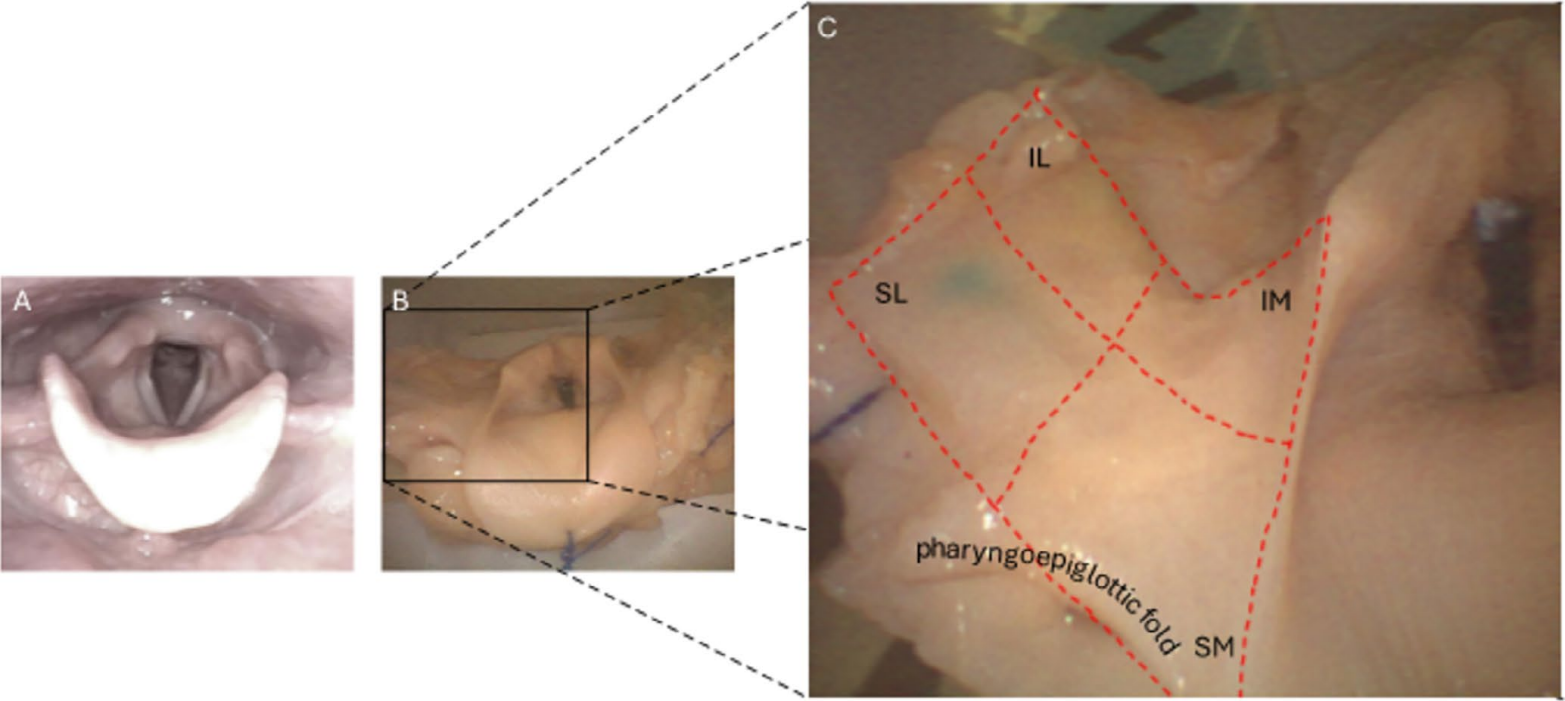
Endoscopic view on the larynx in vivo (A) and within the model (B). (C) Zoom into the right piriform sinus showing the quadrants. IL, inferolateral quadrant; IM, inferomedial quadrant; SL, superolateral quadrant; SM, superomedial quadrant.

A flexible endoscope with a working channel, the aScope 4 RhinoLaryngo (Ambu A/S, Ballerup, Denmark), was introduced through the nasal cavity into the nasopharynx. This setup provided clear visualization of the piriform sinuses, larynx, and the superior border of the thyroid cartilage. As noted in the introduction, we sought to mark the superolateral quadrant of the piriform sinus as defined here. The region of interest in the piriform sinus was conceptually divided into four quadrants. The quadrants are created by the outer boundaries of a rectangle, and the area divided into four smaller rectangles by a horizontal midline and a vertical midline. The large rectangle area is essentially the medial surface of the thyrohyoid membrane region as seen endoscopically (Figure 2). The outer superior boundary is defined by the pharyngoepiglottic fold. The outer inferior boundary is defined by the upper edge of the thyroid cartilage. These two boundaries are approximately parallel to each other. So, the horizontal midline is parallel to and halfway between the two aforementioned boundaries (Figure 2). The vertical midline boundary is defined by the deepest point of the pharyngoepiglottic fold (Figure 2). The outer lateral boundary is the vertical line where the halfpipe of the curved piriform sinus becomes straight and transitions into the lateral pharyngeal wall. The outer medial boundary is the vertical line where the medial halfpipe of the piriform sinus becomes straight as it terminates in the aryepiglottic fold. Halfway between those vertical lines is the vertical midline. The perpendicular intersection of the vertical and horizontal lines creates the four quadrants. Again, targeted ink markings are aimed at the superolateral quadrant.

Toluidine blue ink (Carolina, Burlington, NC, USA) was used to mark the superolateral quadrant of the piriform sinus bilaterally, using an injection needle—the Injector Force Max Injection Needle (NM‐401L‐0425; Olympus, Tokyo, Japan)—via the endoscope's working channel. After marking, the larynx was removed from the model in order to allow for measurements.

### 2.4 Measurements

First, measurements were taken of the thyroid cartilage and its size assessed. Then the iSLN and the superior thyroid artery were dissected to their points of entry into the thyrohyoid membrane. The artery was distinguished from the nerve by the presence of a visible lumen.

**FIGURE 3:**
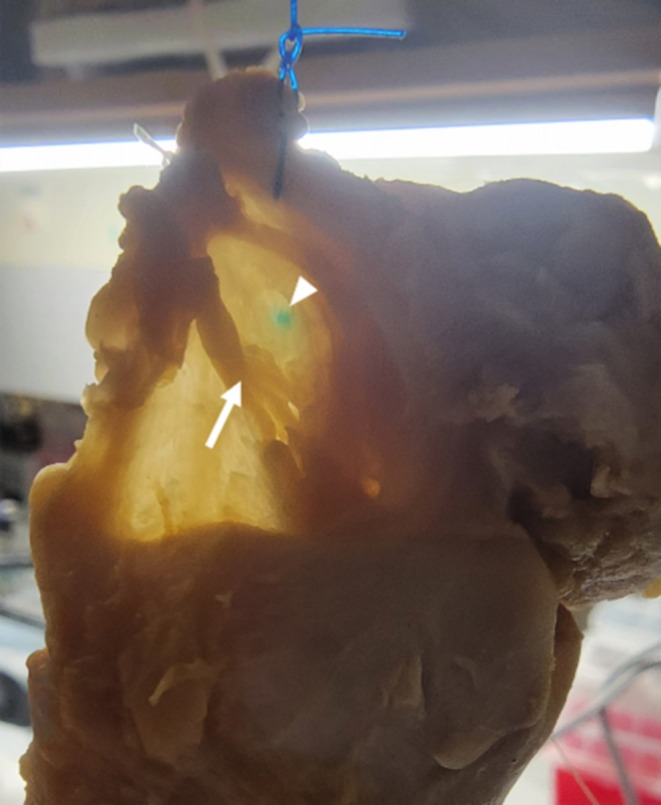
Transillumination of the mucosa using a strong light source (diaphanoscopy) was performed to visualize the anatomical structures without damaging the surrounding tissue. The image illustrates the distance between the confluence point of the superior laryngeal nerve (arrow) and the site of the mucosal ink mark (arrowhead).

Finally, the distance between the ink mark and the confluence point of the iSLN was measured using diaphanoscopy, a technique in which the tissues are transilluminated with a light source to avoid damaging the mucosa (Figure 3).

Measurements on the specimens were taken bilaterally using an electronic digital caliper (01407A; NEIKO, Corona, CA, USA) with a resolution of 0.01 mm and an accuracy of ±0.02 mm.

### 2.5 Statistical Analysis

All statistical analyses were performed using SPSS (Version 28). To determine whether the data were suitable for parametric testing, we first assessed normality using the Shapiro–Wilk test. A *p* value greater than 0.05 in this test indicates that the data do not significantly deviate from a normal distribution.

If normal distribution was confirmed, we used a one‐sample *t*‐test to compare the mean distances from our measurements with the reference data provided by Kiray et al. [11].

To assess whether the variability of the measurements differed across groups, we additionally performed Levene's test. This test checks whether different groups have similar variances in the distances.

A *p* value less than 0.05 was considered statistically significant.

## 3 Results

### 3.1 Specimens

A total of eight specimens were measured bilaterally, resulting in 16 data points for each measurement. In one larynx, a fracture of the superior horn of the thyroid cartilage was observed. In the measurement where the superior horn served as a reference point, the affected side was excluded from the corresponding analysis.

### 3.2 Model

All larynges, regardless of their size variations, were successfully positioned within the model without any difficulty. The nasopharyngeal cavity was slightly oversized, leading to inadequate camera guidance and tilting in the frontal and sagittal planes. To address this, part of the nasopharynx was reconstructed using modeling clay (Play‐Doh, Hasbro Inc.), ensuring realistic and stable camera movements.

### 3.3 Measurements

Measurements assessing laryngeal dimension are shown in Table 1. Four key distances were analyzed statistically, with all measurements confirmed to follow a normal distribution as indicated by the following *p* values: greater horn of the hyoid bone to the confluence point (*p* = 0.622), superior horn of the thyroid cartilage to the confluence point (*p* = 0.839), distance from ink mark to the confluence point (*p* = 0.880), and greater horn of the hyoid bone to the middle branch piercing through the thyrohyoid membrane (*p* = 0.878).

**TABLE 1:** Summarizes the measurements of laryngeal dimensions and the distances between key anatomical structures (mean ± SD [mm]).

Laryngeal prominence to superior horn of thyroid cartilage	Greater horn of hyoid to superior horn of thyroid cartilage	Upper margin of thyroid cartilage to confluence point	Greater horn to middle branch piercing through thyrohyoid membrane	Confluence point to piercing point of the middle branch
47.73 ± 9.83	20.08 ± 6.40	7.35 ± 3.13	25.05 ± 5.32	11.66 ± 3.50

The one‐sample *t*‐test showed no significant difference (*p* = 0.583) between our measurements and the corresponding values reported by Kiray et al. [11].

Levene's tests demonstrated significantly higher variability in the distances from the greater horn of the hyoid bone to the confluence point (*p* = 0.018) and from the superior horn of the thyroid cartilage to the confluence point (*p* = 0.005), compared to the distance from the ink mark to the confluence point (Table 2).

**TABLE 2:** Distances from key anatomical landmarks to the confluence point. Levene's test *p* values (bottom row) compare the variability of the ink–confluence distance with the variability of the greater horn–confluence and superior horn–confluence distances.

	Ink to confluence point	Greater horn to confluence point	Superior horn to confluence point
Mean ± SD (mm)	5.38 ± 1.63	12.28 ± 4.33	9.20 ± 3.56
Levene's Test *p* value	Reference	0.018	0.005

## 4 Discussion

The internal branch of the superior laryngeal nerve (iSLN) appears to transmit the critical pathological signals underlying laryngeal sensory dysfunction (LSD) [12]. LSD is thought to contribute to symptoms such as unexplained chronic cough, chronic throat clearing, globus pharyngeus, and paradoxical vocal fold motion [13, 14]. As such, it is frequently implicated in the diverse and often debilitating symptoms reported by patients with this condition. Among these, chronic cough has shown particularly favorable responses to local anesthetic injections targeting the iSLN [3]. However, presumably due to considerable anatomical variability among individuals, external approaches to the nerve often lack the precision required for consistent and effective blockade, which may explain the reported effect duration of only about two months [4, 7] and the need in certain cases for multiple weekly injection [3]. Clinically, our group has transitioned largely to using a transnasal technique for injection [8]. Briefly, the course of the iSLN has been described previously, and neuromodulation of the iSLN with therapeutic medicine was first described in 1911 [15], meaning this concept is not new. The anatomic examinations of the larynx show that there are three nerves (inferior, middle, and superior) that become confluent as the iSLN as it leaves the pharynx through the thyrohyoid membrane [10]. The transnasal technique leverages known anatomy and the typically visible presence of the iSLN as it moves laterally out towards the thyrohyoid membrane. Based upon our observations that it moves superiorly as it moves laterally, we have chosen the superolateral quadrant—described above—as our target area when injections are performed clinically. However, to increase confidence in anatomical assumptions, we aimed to validate the presumed location of the iSLN and propose the quadrant concept that all clinicians can apply to improve the accuracy of their injections.

To study this challenge, we used a novel, anatomically guided technique using a hybrid 3D head/neck model to access the iSLN via the mucosa of the piriform sinus. This internal approach has the potential to provide a more reliable path to the nerve, minimizing variability and improving targeting accuracy.

### 4.1 Validation of the Measurements

To provide a detailed anatomical description of the iSLN, Kiray et al. [11] analyzed 24 formaldehyde‐fixed adult larynges, documenting various parameters such as its length and relationship to adjacent structures. Among these parameters was the distance from the greater horn of the hyoid bone to the middle branch piercing the thyrohyoid membrane, which they reported as 25.8 ± 5.5 mm on average. In our study, the corresponding average measurement was 25.05 ± 5.32 mm, with no significant difference between the two values in a one‐sample *t*‐test. This comparison suggests the accuracy of our distance measurements.

While the confluence point of the superior laryngeal nerve branches lies laterally to the thyrohyoid membrane, this finding does not substantially limit the feasibility of an internal approach via the piriform sinus. Given the maintained proximity of the nerve branches, targeted access remains achievable. Introducing an internal technique alongside established external approaches enhances the physician's therapeutic options for treating laryngeal sensory dysfunction, allowing for greater adaptability to anatomical variations and patient needs.

### 4.2 Therapeutic Approaches to the iSLN

The iSLN has historically been a focus of therapeutic interventions, particularly during the early 20th century when laryngeal tuberculosis necessitated innovative solutions for symptom management [16]. Patients had tubercular nerve invasion with accompanying odynophagia, and alcohol was injected transorally into the region of the nerve in the piriform sinus [9, 17]. More recently, the superior laryngeal nerve block has been employed to alter laryngeal sensitivity and facilitate awake endotracheal intubation by injecting a local anesthetic near the nerve's passage through the thyrohyoid membrane [18]. Common injection sites include the thyroid cartilage and the hyoid bone. However, this approach is rarely used in clinics due to its inconsistency, primarily caused by difficulties in palpating the reference structures [19].

A case report demonstrated the use of ultrasound guidance to inject lidocaine near the hyoid bone when it was not palpable, facilitating awake intubation [20]. Similarly, this technique has been shown to effectively reduce post‐extubation sore throat in a randomized controlled trial [21]. Although more reliable, the approach might remain challenging in patients with short necks and obesity, highlighting the limitations and complexities of these external techniques.

In laryngology, anesthesia of the iSLN has gained attention in managing laryngeal sensory dysfunction (LSD), which can present as globus pharyngeus, allotussia, or hypertussia and possible paradoxical vocal fold motion [12]. A retrospective case series demonstrated the efficacy of a mixture of triamcinolone acetonide, lidocaine, and epinephrine in improving the Cough Severity Index among 10 patients with neurogenic cough. In this study, the thyroid cartilage and hyoid bone served as palpatory landmarks for injection [22].

Novakovic et al. described an endoscopic, trans‐thyrohyoid approach to inject botulinum toxin into the supraglottic region for LSD patients. Outcomes assessed via the Newcastle Laryngeal Hypersensitivity Questionnaire, Cough Severity Index, Reflux Symptom Index, and Voice Handicap Index‐10 (VHI10) showed significant improvements in all measures except the VHI10 after botulinum toxin administration [12].

These studies collectively underscore the therapeutic potential of neuromodulatory injections for LSD, targeting the iSLN where it passes through the thyrohyoid membrane. This location is likely chosen because it is presumed to be close to the nerve's confluence point, enabling access to all branches of the iSLN.

Despite these promising results, current methods for anesthetizing the iSLN rely heavily on external landmarks, that is, thyroid cartilage and hyoid bone. Anatomical variability among patients often leads to inconsistent outcomes [20, 23]. To address these limitations, our previous study [8] introduces an alternative approach: accessing the iSLN via the mucosa of the piriform sinus. This method eliminates the reliance on external landmarks, offering a potentially more reliable injection site. Our data support this hypothesis, as Levene's test demonstrated significantly lower variability in the distance from the proposed mark site to the confluence point compared to external landmarks. This reduced variability suggests a higher reliability of the injection when using our proposed method. A mean distance of about 5 mm from the mark point to the confluence of the iSLN confirms our hypothesis that the superolateral quadrant of the piriform sinus is an adequate choice for the delivery of pharmacotherapeutics since diffusion of even small amounts of liquid medication should diffuse adequately.

Alternative approaches, such as injections via the vallecula, were deemed unsuitable due to the proximity of the superior laryngeal artery, which increases the risk of vascular complications or airway obstruction. Additionally, anatomical variations such as a hypertrophic tongue base further limit accessibility in certain patients [24].

### 4.3 Limitations

A key limitation of this model is the use of cadaveric larynges, which lack the dynamic movements present in living tissue. Another limitation is the midline nasal access used in the model, which differs slightly from the unilateral approach typically used in clinical practice. This discrepancy is unlikely to pose a significant challenge based on the authors' clinical expertise.

## 5 Conclusion

The method proposed in this study offers a novel and potentially more consistent approach for accessing the iSLN, paving the way for improved therapeutic interventions in patients with LSD. Future research, including randomized controlled trials, will be crucial to validate these findings and establish this technique as a standard of care.

## Conflicts of Interest

The authors declare no conflicts of interest.

## Data Availability

The data that support the findings of this study are available from the corresponding author upon reasonable request.
